# Pathogenic MAX Variant in Bilateral Adrenal Paragangliomas: The Dilemma of Cortical-Sparing Surgery

**DOI:** 10.7759/cureus.97541

**Published:** 2025-11-23

**Authors:** Henrique Carmona Alexandrino, Andreia Martins Fernandes, Ricardo Godinho, Noémia Castelo Branco, Raquel Martins

**Affiliations:** 1 Endocrinology, Unidade Local de Saúde Gaia-Espinho, Vila Nova de Gaia, PRT; 2 Endocrinology, Instituto Português Oncologia de Coimbra Francisco Gentil, Coimbra, PRT; 3 Urology, Instituto Português Oncologia de Coimbra Francisco Gentil, Coimbra, PRT; 4 Pathology, Instituto Português Oncologia de Coimbra Francisco Gentil, Coimbra, PRT

**Keywords:** adrenal paraganglioma, bilateral adrenalectomy, cortical-sparing adrenalectomy, hereditary paraganglioma-pheochromocytoma syndromes, max gene

## Abstract

We describe the case of a woman in her late 30s presenting with signs and symptoms of catecholamine excess and asymmetric bilateral adrenal lesions. Initial genetic testing was negative, including analysis of RET, VHL, SDH genes, TMEM127, and MAX. She underwent right cortical-sparing adrenalectomy, which normalized biochemical markers and blood pressure. One year later, she developed recurrent catecholamine excess, and repeat genetic analysis identified a germline pathogenic variant in MAX. Functional imaging confirmed a contralateral adrenal lesion, raising considerations regarding oncological safety versus adrenal preservation. This case highlights the complexity of hereditary pheochromocytoma/paraganglioma syndromes and illustrates how evolving genetic testing can influence surgical decision-making.

## Introduction

Paragangliomas (PGLs) are rare non-epithelial neuroendocrine neoplasms (NENs) arising from autonomic paraganglia. Since the 2022 WHO Classification of Endocrine and Neuroendocrine Tumors (5th Edition), pheochromocytomas (previously distinguished from paragangliomas) are now designated as adrenal paragangliomas (aPGLs), given their origin in the adrenal medulla, the largest sympathetic paraganglion [1]. Extra-adrenal PGLs arise from sympathetic or parasympathetic paraganglia along the autonomic nervous system. These tumors commonly secrete catecholamines, including norepinephrine, epinephrine, or, less frequently, dopamine, and have an incidence of approximately 0.58 per 100,000 person-years [2,3].

Genetics play a pivotal role in PGL, with up to 40% of cases attributed to germline mutations [4]. PGLs can be categorized into three molecular clusters with different characteristics: pseudohypoxia-associated (cluster 1), kinase signaling-associated (cluster 2), and Wnt signaling-associated (cluster 3) [5].

This case involves a patient with bilateral aPGLs in the context of a MAX mutation. Management of MAX-related PGLs is challenging due to their multifocal nature, frequently bilateral, malignancy potential, and recurrence risk. While total bilateral adrenalectomy has been the standard treatment, cortical-sparing adrenalectomy offers adrenal preservation but with a higher risk of recurrence. This case highlights the complexities of surgical decision-making in hereditary aPGL, underscoring the importance of individualized approaches that balance oncological safety, adrenal preservation, and patient preference.

## Case presentation

A woman in her late 30s, with no significant family history of hypertension or malignancy, presented to the emergency department with a hypertensive crisis. She described a year-long history of episodic paroxysmal hypertension, hyperhidrosis, and headaches. Her medical history included a 10-year diagnosis of hypertension, managed with telmisartan and amlodipine. At presentation, her blood pressure was markedly elevated (220/150 mmHg), with mild tachycardia (116 beats per minute). Physical examination revealed no signs of target organ damage or syndromic features. She was admitted for blood pressure control and discharged with a recommendation for further outpatient evaluation.

**Figure 1:**
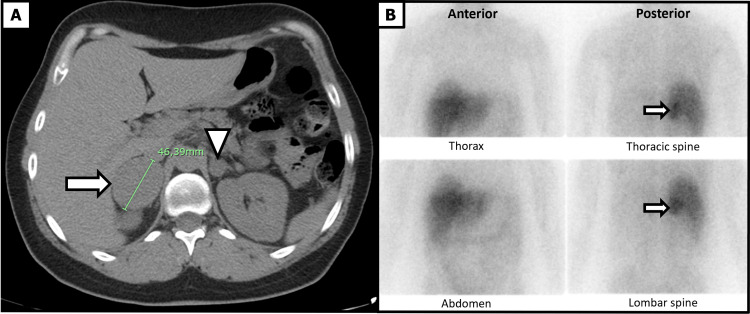
Bilateral adrenal lesions on CT and MIBG scintigraphy A) Axial CT scan showing a right adrenal lesion (horizontal white arrow) and a partially visualized left adrenal lesion (arrowhead). B) Iodine-131-MIBG scintigraphy demonstrating intense focal uptake in the right adrenal lesion (horizontal white arrow). On the left side, only a very faint, low-grade activity was observed in the expected adrenal topography, with intensity markedly lower than hepatic background, and interpreted as likely physiological adrenal uptake rather than evidence of a focal lesion. MIBG - metaiodobenzylguanidine

The initial diagnostic work-up (Table 1) revealed significantly elevated plasmatic normetanephrines and slightly elevated plasmatic metanephrines, consistent with a strong biochemical suspicion of an adrenal paraganglioma. An abdominopelvic CT scan demonstrated bilateral adrenal lesions: a 42 mm hyperenhancing mass in the right adrenal gland, described as radiologically compatible with an adrenal paraganglioma, and a 15 mm enhancing left adrenal nodule that lacked typical features of an adenoma. Although quantitative parameters such as Hounsfield units and contrast washout values were not reported in the externally performed scan, the adrenal multidisciplinary team reviewing the images concluded that neither lesion exhibited imaging characteristics suggestive of an adenoma (Figure 1).

**Table 1:** Initial diagnostic work-up TSH - thyroid stimulating hormone; ACTH - adrenocorticotropic hormone

	Admission	Reference range
1^st^ line workup		
Hemoglobin (g/dL)	13.7	13.0 – 18.0
Creatinine (mg/dL)	0.8	0.5 – 1.2
Ionized calcium (mmol/L)	1.19	1.14-1.29
Glucose (mg/dL)	86	70 – 115
Sodium (mmol/L)	137	135 – 150
Potassium (mmol/L)	4	3.5 – 5.3
Parathyroid hormone (pg/mL)	57	15-65
TSH (mUI/L)	2.63	0.45-4.50
Plasmatic metanephrine (pg/mL)	167	<65
Plasmatic normetanephrine (pg/mL)	3679	<196
Plasmatic 3-metoxityramine (pg/mL)	80.5	<175
2^nd^ line workup		
Cortisol (μg/dL)	13.8	6.7-22.6
ACTH (pg/mL)	18.3	7.2-63
Cortisol post 1mg dexamethasone (μg/dL)	1.7	<1.8
Dehydroepiandrosterone sulfate (μg/dL)	46.9	23-266
Androstenedione (ng/mL)	0.59	0.3-3.3
17-Hydroxyprogesterone (ng/mL)	1.1	0.2-2.5
Calcitonin (pg/mL)	0.8	<9.8
Chromogranin A (ng/mL)	1219	<39

The findings were reviewed during a multidisciplinary tumor board meeting. The board recommended an urgent germline genetic analysis for familial syndromes associated with PGL, including RET, VHL, SDHx, TMEM127, and MAX. An iodine-131-metaiodobenzylguanidine (I-131 MIBG) scintigraphy was performed, confirming intense uptake in the right adrenal lesion and minimal uptake in the left adrenal gland (Figure 1).

A second board meeting reviewed the imaging and genetic findings, which were negative for germline mutations. Based on the biochemical, genetic, and radiological findings, a staged approach was proposed, beginning with cortical-sparing resection of the larger right-sided lesion, to manage the right-sided aPGL while preserving adrenal function, with the final approach contingent on intraoperative findings and subsequent histopathological results. Preoperatively, the patient was initiated on phenoxybenzamine (10 mg twice daily, titrated to 30 mg three times daily) and propranolol (10 mg three times daily) for effective alpha- and beta-blockade. This regimen successfully stabilized her blood pressure and symptoms, enabling surgical planning.

Treatment

The patient underwent a right cortical-sparing adrenalectomy, which successfully removed a 5 cm mass from the right adrenal gland. Postoperatively, her blood pressure stabilized within the low-normal range, allowing for the discontinuation of phenoxybenzamine and propranolol. Six weeks after surgery, plasma and urinary levels of metanephrines and normetanephrines normalized.

**Figure 2:**
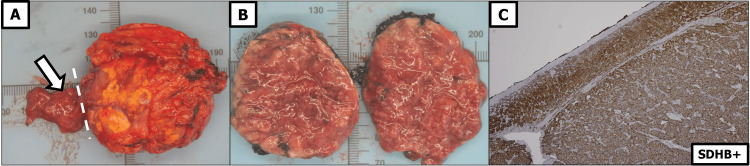
Gross and Microscopic Features of the Right Adrenal Paraganglioma Panel A: Right adrenal mass measuring 50 mm; white arrow indicates residual normal adrenal gland tissue. Panel B: Cut surface of the adrenal paraganglioma (aPGL), demonstrating high vascularity. Panel C: Immunohistochemistry highlighting granular cytoplasmic positivity for succinate dehydrogenase subunit B (SDHB+).

Histopathological analysis confirmed the diagnosis of aPGL. Tumor cells showed strong immunoreactivity for chromogranin A, CD56, and synaptophysin, with a Ki-67 proliferation index of 4%. SDHB expression was granular and cytoplasmic, and the tumor was staged as pT3 (Figure 2).

Given the normalization of metanephrine levels and the absence of blood pressure abnormalities without medication, adrenalectomy of the left adrenal gland was deemed unnecessary at the time. A follow-up CT scan five months later revealed a small 9 mm nodule in the right adrenal bed and an 18 × 15 mm lesion in the left adrenal gland, which remained stable. Based on the available information-including negative initial genetic testing, resolution of symptoms after right adrenalectomy, radiological stability, and the previously described very faint MIBG activity - the left adrenal lesion was initially considered most likely an adrenal adenoma. However, a contralateral, non-secretory adrenal paraganglioma could not be entirely excluded. The patient continued under clinical observation and remained asymptomatic for one year.

Outcome and follow-up

One year after surgery, the patient experienced recurrence of symptoms suggestive of catecholamine excess, including new-onset hypertension, headaches, and hyperhidrosis. Biochemical testing revealed a threefold elevation of plasma and urinary normetanephrines above the upper limit of normal, and we reinitiated phenoxybenzamine. An urgent genetic reanalysis identified a heterozygous PV in the MAX gene (c.223C>T, p.(Arg75*)). Functional imaging with positron emission tomography and computed tomography (PET/CT) using l-6-(18F)fluoro-3,4-dihydroxyphenylalanine (18F-DOPA) demonstrated anomalous uptake in a 10 × 8 mm nodular formation in the right adrenal surgical bed, as well as significant uptake in the left adrenal gland lesion. These findings were consistent with recurrent and contralateral aPGL.

**Figure 3:**
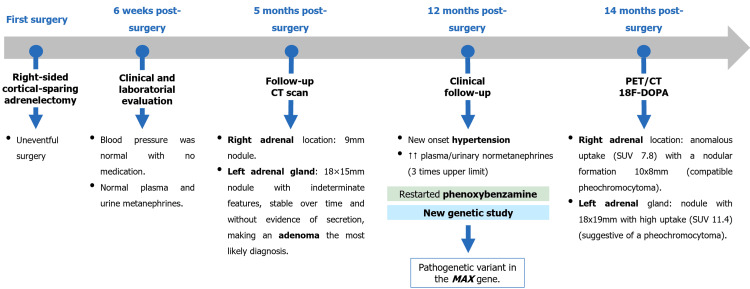
Timeline following the first surgery

The case was revisited by the multidisciplinary tumor board, and a surgical approach was favored given the presence of localized small-moderate volume disease. In particular, the lesion in the right adrenal surgical bed was considered technically difficult to resect due to its small size and prior surgical intervention. For the left adrenal lesion, the board extensively debated the oncological safety of total adrenalectomy versus cortical-sparing adrenalectomy, considering the MAX PV malignancy risk, recurrence potential, and the patient's quality of life. After reviewing the risks and benefits, the patient opted for a left cortical-sparing adrenalectomy to avoid lifelong steroid dependence (Figure 3). 

**Figure 4:**
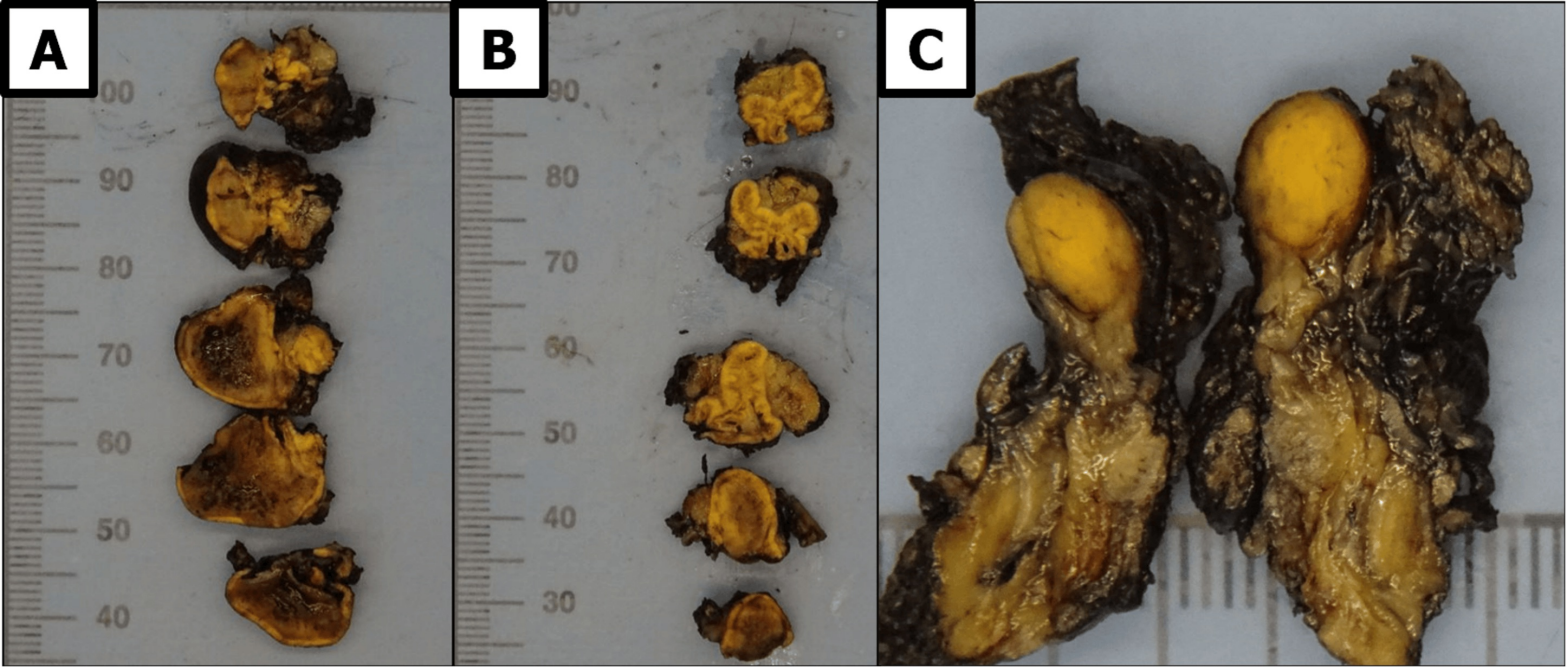
Macroscopic appearance of multifocal adrenal paraganglioma of the left adrenal gland A) The largest nodular lesion, measuring 25 mm. B, C) two additional nodular formations, measuring 9 mm and 8 mm, respectively.

Intraoperatively, hydrocortisone (100 mg) was administered to mitigate the risk of adrenal insufficiency. The surgery proceeded without complications, and postoperative morning cortisol levels exceeded 18 μg/dL, confirming preserved adrenal function without the need for steroid replacement. Blood pressure normalized without any medication. Histopathological examination of the left adrenal gland revealed multifocal aPGL, with three foci measuring 25 mm, 9 mm, and 8 mm. The Ki-67 index was 3%, and the tumor was staged as pT1(m) (Figure 4).

Three months postoperatively, follow-up abdominal MRI showed a stable pericentimetric lesion in the right adrenal surgical bed and no evidence of recurrence in the left adrenal gland. At her most recent follow-up, two years after the second surgery, the patient remains asymptomatic with normal blood pressure and no biochemical evidence of recurrence. She continues regular surveillance with biannual imaging and biochemical screening for aPGL recurrence, as well as screening for primary hyperparathyroidism and other manifestations associated with the MAX PV. Genetic testing of her parents was negative for the MAX mutation, suggesting a de novo pathogenic variant. Screening of her offspring has been deferred due to their minor status and the fact that MAX shows a paternal parent-of-origin effect.

## Discussion

**Figure 5:**
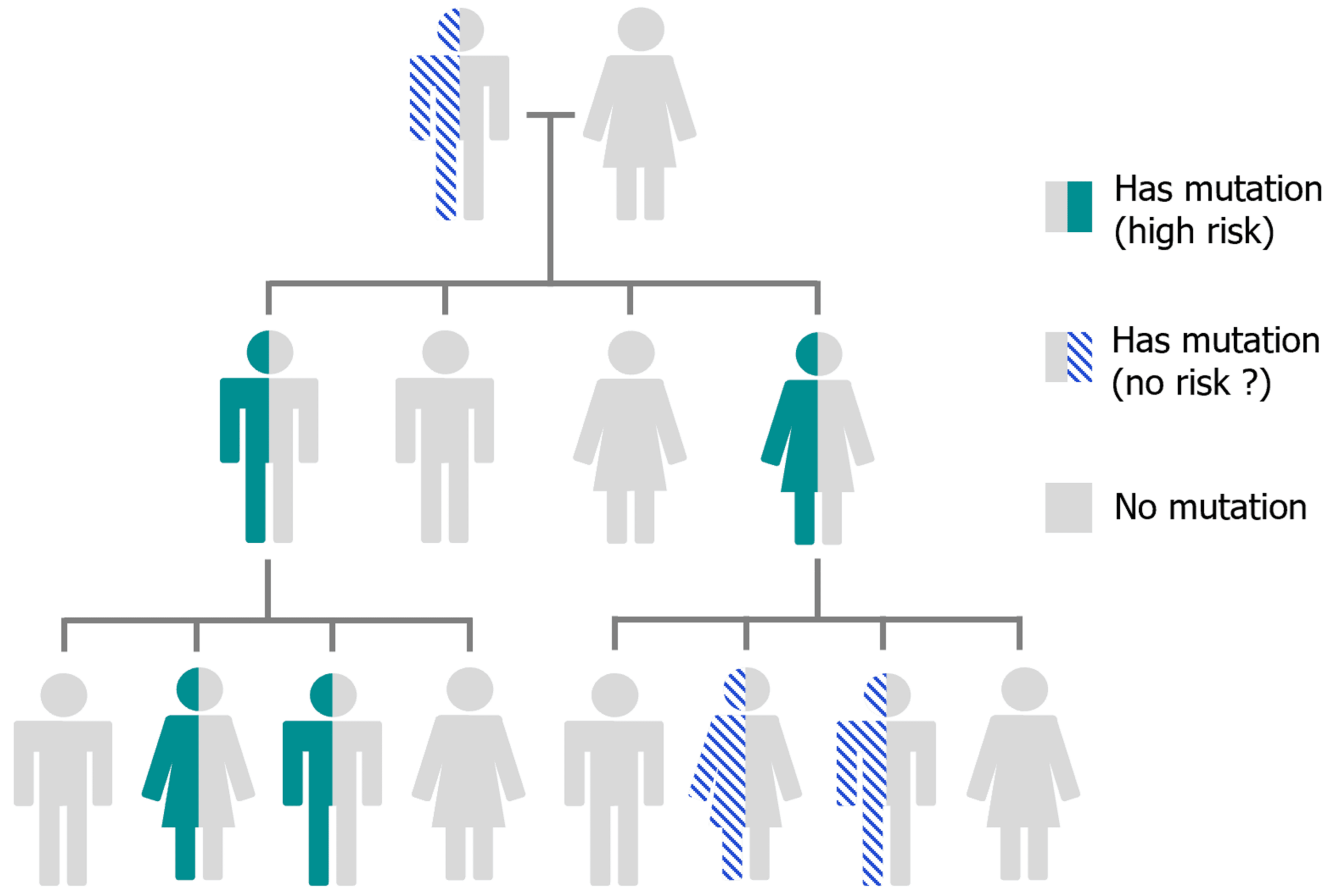
Pedigree illustrating autosomal dominant inheritance of MAX pathogenic variants Pedigree illustrating autosomal dominant inheritance of MAX pathogenic variants (PVs), with a 50% chance of transmission to offspring. Although autosomal dominant, maternal imprinting has been suggested but is not yet fully confirmed due to the rarity of reported cases. To date, no disease manifestation has been described following maternal transmission. If maternal imprinting is definitively established, individuals who inherit the mutation from their mother may not be at risk and would not require clinical follow-up. In contrast, those who inherit the mutation from their father are considered at high risk and require surveillance. The term "no risk?" in the legend reflects the current lack of sufficient evidence to draw firm conclusions. Original illustration created by the authors.

The diagnosis of a hereditary PGL syndrome should be considered in all patients with PGL but is highly suspected in individuals with multiple, multifocal, recurrent, or early-onset PGL [6]. This was the case of our patient, who presented with an aPGL at a young age. The myelocytomatosis (MYC)-associated factor X (MAX) encodes the MAX protein. MYC is a proto-oncogene family that has an essential role in cell cycle regulation, differentiation, and is thought to have mostly maternal imprinting (i.e., tumor formation occurs almost exclusively through paternal transmission (Figure 5) [7].

In 2021, this gene was proposed to constitute a new multiple endocrine neoplasia syndrome, provisionally termed MEN5, and a 2024 review of over 100 reported patients recommended screening for PGL, primary hyperparathyroidism, ganglioneuroma/ neuroblastoma, and pituitary adenomas [8,9]. It is recommended that these patients should be screened with cross-sectional imaging of the skull base to pelvis every three to five years and annual clinical review and biochemistry [10].

**Figure 6:**
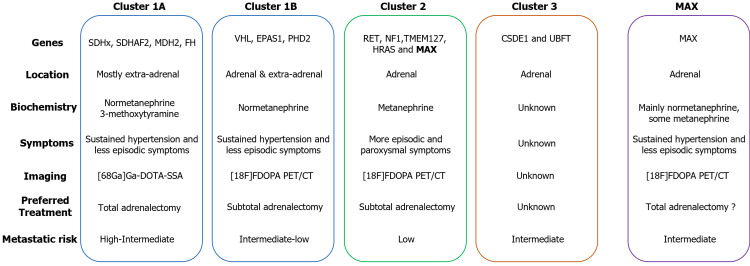
Summary of the main molecular clusters of PGL and their associated features Preferred treatment reflects general recommendations, but management should be individualized based on clinical context. Metastatic risk is comparative and varies among individual genes. (68Ga)Ga-DOTA-SSA: 68 Ga-DOTA-somatostatin analog (SSA); (18F)FDOPA PET/CT: 3,4-dihydroxy-6-18F-fluoro-L-phenylalanine. PGL - paraganglioma Original illustration created by the authors.

MAX pathogenic variants (PVs) are mostly associated with bilateral aPGL, are part of cluster 2, and encompass around 1-4% of all PGL cases (Figure 6) [6,11].

This cluster is usually associated with almost exclusively located aPGL, with mostly adrenergic phenotype (i.e., predominantly elevations of metanephrines compared to normetanephrines), with signs and symptoms mostly episodic and paroxysmal. The overall metastatic risk in this group is considered to be low (around 2%-4%) [12]. This has sparked some debates regarding the extent of surgical procedure, as total bilateral adrenalectomy has been a standard treatment, but it has a 100% risk of adrenal insufficiency [13]. As an alternative surgical approach, cortical-sparing adrenalectomy reduces this risk to 25% but increases the risk of local recurrence [14]. However, the PRAP study showed that the metastasis rate or disease-specific mortality did not differ between the two approaches [15].

**Figure 7:**
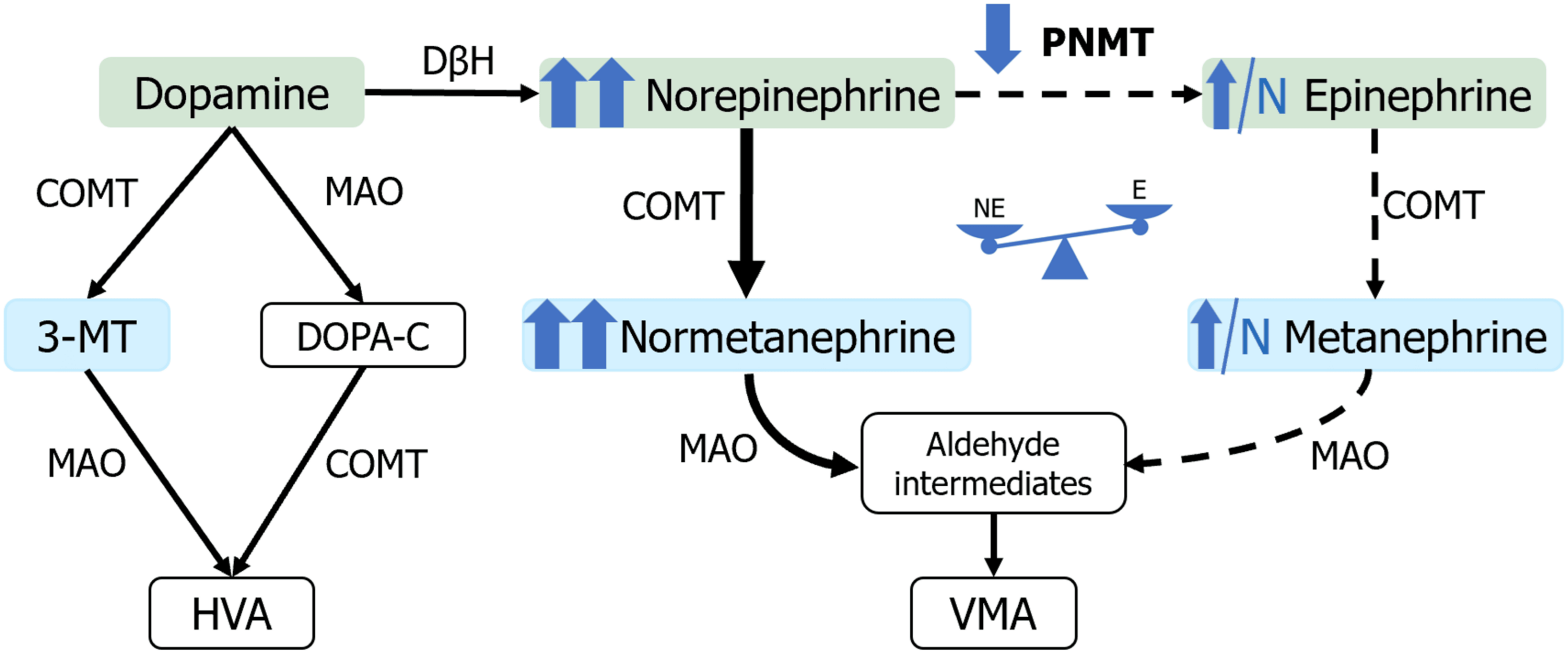
Biochemical phenotype associated with MAX pathogenic variants (PVs) Decreased (but not absent) expression of PNMT leads to a disproportionate increase in norepinephrine and, consequently, normetanephrine levels. However, some production of metanephrines may still occur in certain cases. 3-MT - 3-methoxytyramine; HVA - homovanillic acid; VMA - vanillylmandelic acid; NE - norepinephrine; E - epinephrine Original illustration created by the authors.

However, MAX PVs are unique within their own cluster due to a few key differences. First, they have an intermediate biochemical phenotype that secretes predominantly norepinephrine compared to epinephrine, which has mixed symptoms and signs (sustained hypertension and paroxysms), which was the case of our patient who presented with long-standing hypertension and later with paroxysms [16]. This is mainly due to different mRNA expression of phenylethanolamine N-methyltransferase (PNMT) gene (see Figure 7) [16].

This may also explain its somewhat inconsistent uptake of 123-MIBG at different adrenal lesions as seen with other patients with MAX PVs [17]. Secondly, MAX-related aPGLs appear to have a higher malignant potential than initially assumed: earlier series suggested a metastatic risk of approximately 10%, whereas the PRAP study reported a higher rate of around 25%, albeit based on a very limited sample size [15]. This observation is consistent with broader analyses indicating that MAX belongs to the subgroup of susceptibility genes associated with increased recurrence and metastatic potential [18]. Such considerations may limit the oncological safety of cortical-sparing adrenalectomy. At the time we proposed the second surgery, the literature remained scarce regarding the metastatic risk and long-term oncological safety of cortical-sparing adrenalectomy in MAX-associated disease. After a very thorough discussion with the patient and considering the information we had at that time, her choice was to undergo a partial surgery. This case highlights the complexity of managing aPGL associated with MAX PVs, given the limited number of reported cases. Considering the emerging evidence of non-negligible metastatic risk, high penetrance, and multifocality of the disease, cortical-sparing adrenalectomy may be less advantageous for patients carrying MAX PVs.

## Conclusions

This case highlights the importance of an individualized approach to the surgical management of bilateral adrenal paragangliomas, particularly in hereditary syndromes associated with MAX pathogenic variants, where oncological safety must be carefully balanced against the risk of lifelong adrenal insufficiency. Cortical-sparing adrenalectomy offers a valuable alternative to total adrenalectomy by preserving adrenal function and avoiding lifelong steroid dependence; however, it carries an increased risk of local recurrence and therefore requires diligent, long-term surveillance.

Importantly, this case also illustrates that in carefully selected patients, such as those with small-volume disease, absence of metastasis, and strong preference to avoid lifelong steroid therapy, bilateral cortical-sparing adrenalectomy may be feasible and can lead to good short-term outcomes, as demonstrated by our patient's preserved adrenal function and absence of recurrence at two years.

Finally, the case underscores the key role of multidisciplinary tumor boards in guiding decision-making, integrating endocrine, surgical, genetic, and radiological expertise to tailor treatment strategies to both patient preference and current clinical evidence.
